# Expression of Lectins in Heterologous Systems

**DOI:** 10.3390/ijms19020616

**Published:** 2018-02-21

**Authors:** Dania Martínez-Alarcón, Alejandro Blanco-Labra, Teresa García-Gasca

**Affiliations:** 1 Departamento de Biotecnología y Bioquímica, Centro de Investigación y Estudios Avanzados del IPN, Km. 9.6 Libramiento Norte, Carretera Irapuato-León, Irapuato 36824, Guanajuato, Mexico; dania_667@hotmail.com (D.M.-A.); alejandroblancolabra@gmail.com (A.B.-L.); 2 Facultad de Ciencias Naturales, Universidad Autónoma de Querétaro, Av. de las Ciencias s/n, Juriquilla, Santiago de Querétaro 76230, Querétaro, Mexico

**Keywords:** lectins, recombinant proteins, heterologous systems

## Abstract

Lectins are proteins that have the ability to recognize and bind in a reversible and specific way to free carbohydrates or glycoconjugates of cell membranes. For these reasons, they have been extensively used in a wide range of industrial and pharmacological applications. Currently, there is great interest in their production on a large scale. Unfortunately, conventional techniques do not provide the appropriate platform for this purpose and therefore, the heterologous production of lectins in different organisms has become the preferred method in many cases. Such systems have the advantage of providing better yields as well as more homogeneous and better-defined properties for the resultant products. However, an inappropriate choice of the expression system can cause important structural alterations that have repercussions on their biological activity since the specificity may lay in their post-translational processing, which depends largely on the producing organism. The present review aims to examine the most representative studies in the area, exposing the four most frequently used systems (bacteria, yeasts, plants and animal cells), with the intention of providing the necessary information to determine the strategy to follow in each case as well as their respective advantages and disadvantages.

## 1. Introduction

Lectins are proteins that include at least one non-catalytic domain with the ability of binding in a reversible and specific way to carbohydrates that are free or bound to cell membranes, such as glycoproteins, glycolipids or polysaccharides [1,2,3]. The recognition of these carbohydrates is highly specific and allows these proteins to differentially bind to certain molecules and cells [4,5]. Due to this selectivity, lectins have been widely used for the investigation of complex carbohydrates and changes occurring in cellular interactions during physiological and pathological processes in recent years, providing evidence for a wide spectrum of industrial and pharmacological applications. Therefore, there is an increasing interest both in their purification and in their characterization. Chromatographic techniques have been most widely used for the purification of lectins from their natural sources. However, such techniques usually provide very low yields and they are occasionally unable to discriminate between isoforms of the same lectin. Therefore, they are obtained as mixtures, which provides a large range of uncertainty. In this sense, the production of recombinant lectins by genetic engineering techniques has the advantage of providing single proteins, with better yields that allow for their precise characterization [6,7].

There are many reports on the production of recombinant lectins, particularly of plant and animal origin, since they have been shown to have a broader spectrum of applications. Yeasts and bacteria are the preferred system used to express these lectins. However, recombinant lectins have also been expressed in plants and mammalian cells, although this occurred less frequently. The heterologous production of lectins in microorganisms offers simple systems that provide good yields. Unfortunately, such products are often not soluble or not properly processed. In an attempt to circumvent these disadvantages, a number of alternatives have emerged, ranging from refolding [8,9,10], the inclusion of solubilizing peptides [11,12] and the modification of the microorganism glycosylation patterns [13]. Some of these modifications include the expression of human enzymes in yeast strains of *Pichia pastoris*, which are commercially available. On the other hand, mammalian and plant cells are more complex systems that offer a more complete spectrum of post-translational modifications. However, their manipulation is considerably more difficult and costly than yeast and bacteria [14].

Each system presents advantages and disadvantages. The success depends on the analysis of different factors, including the producing organism, and the characteristics of the lectin to be expressed. In this present study, we present the most representative studies in the field of the production of recombinant lectins and link this data through an analysis that aims to contribute to determining the best strategy to follow in each case.

## 2. Synthesis and Processing of Lectins

Lectins can be found in all kingdoms: animalia, plantae, fungi, protista, archea, bacteria and also viruses [5,15]. Their biological functions are highly variable and depend on the organism of origin. For example, it is known that lectins in plants play an important role during nodulation [16] and as part of the defence mechanisms against insects and pathogens [17]. In animals, they are involved in processes of cell migration and adhesion as well as in the control of glycoprotein production and phagocytosis [18]. Furthermore, in bacteria, viruses, fungi and parasites, they are better known as adhesins and their main function is related to the adhesion and colonization processes [19]. Some toxins produced by microorganisms have been considered as possibly belonging to the family of lectins, since they have a recognition domain for carbohydrates (CRD) that serves as an anchor to their target, to then exert their toxic actions. However, these molecules do not satisfy the lectin requirements according to the definitions of Franz and Goldstein [20,21].

Bacteria are relatively simple in their protein synthesis because they lack cellular organelles specialized in post-processing complex proteins and thus synthesize all of their proteins in the cytosol [22]. On the other hand, eukaryotes are significantly more complex. As in prokaryotes, all their proteins, except those synthesized in mitochondria and chloroplasts, begin to be synthesized by free ribosomes in the cytoplasm. Some proteins contain a sequence called “signal peptide”, which is recognized by a signal recognition particle (SRP). The SRP stops the translation and leads the ribosome/protein complex to the external side of the endoplasmic reticulum (ER) membrane. At this point, the complex is recognized by the SRP receptor, which forms a channel through which the protein is introduced. Once this has happened, translation resumes and the protein is directed towards the ER lumen, where the “signal peptide” is cleaved [23,24]. This is the case for most animal and plant lectins. However, in some cases, such as in the lectins from *Nicotiana tabacum* and *Galanthus nivalis*, they are directly synthesized in the cytosol and therefore contain neither peptide signal nor glycosylations [15].

There are considerable differences between eukaryotic lectins synthesized in ER and those that have been synthesized in the cytoplasm. First, the ER lumen is a highly oxidizing environment that favours the formation of disulphide bonds (S–S), while the cytosol maintains the cysteine residues in their reduced form (–SH) [9,25]. On the other hand, the ER lumen contains accessory proteins involved in proper protein folding [23] and has the necessary proteases to cleave the lectin precursors that need to be processed to their functional form, such as in the case of ConA from *Canavalia ensiformis* and favina from *Vicia faba* [25]. In addition, glycosylation takes place in the ER and Golgi apparatus and only lectins with the corresponding signal peptide may present glycosidic antennae. These lectins are particularly resistant to temperature and pH changes due to the attached carbohydrates and the disulphide bonds [26]. Simple forms of glycosylation have also been identified in bacteria [27].

It is important to note that not all lectins synthesized in ER are necessarily glycosylated. For *N*-glycosylation to occur, a primary oligosaccharide of 14 residues (2*N*-acetylglucosamines, three glucoses and nine mannoses) must first be transferred to a sequence of Asn–X–Ser–Thr, where X can be any amino acid except proline [14,28]. This sequence is better known as “Sequon” [28]. Once the proteins have been properly processed in ER, they are embedded in vesicles, which are transported to the Golgi apparatus, where the editing of *N*-glycosidic antennae continues. On the other hand, the *O*-glycosylation process requires the presence of non-phosphorylated serine or threonine residues and also depends on the structural conformation of the already-folded protein [14].

## 3. Classification of Lectins

Lectins are a very heterogeneous group of proteins, with the ability to selectively bind carbohydrates [29]. They have been classified based on different criteria, such as taxonomic origin, cellular localization, specificity of carbohydrate recognition, functions and structure [30]. For the majority, there are well-defined parameters that allow lectins to be considered in all living beings. However, classifications based on structure are usually established for a defined set of organisms, such as plant lectins [31] and animal lectins [32]. Table 1 lists examples of some of these classifications.

## 4. Carbohydrate Recognition

Lectins have complex structures that may or may not include prosthetic groups, such as ions and glycosylations, but their carbohydrate binding capacity is essentially attributed to a typically globular domain termed the “carbohydrate recognition domain” (CRD), which is defined by a conserved group of residues that determine its conformation and function [29]. At times, they also require divalent cations, which form ligand coordination bonds or help to stabilize the CRD by binding the adjacent amino acid [35]. The adjacent domains to the CRD and their non-protein components (if any) can influence the final structure of the protein and its ability to recognize carbohydrates [6,35,36].

In addition to lectins, many other proteins have the ability to interact with carbohydrates, including enzymes, antibodies, some transporters and periplasmic proteins from bacteria. These proteins differ from lectins because their CRDs are deep clefts and they bind with a high affinity that is typically in the order of sub-micromolar dissociation constants. In contrast, lectin CRDs tend to be shallow indentations with lower affinities that are usually of the millimolar order. The selectivity of the CRD is given by combining hydrogen bonds with the hydroxyl groups of the sugar and a Van der Waals packing, which often includes the coverage of a hydrophobic side of the sugar against the side chains of aromatic amino acids. This affinity may be dramatically increased with the pooling of individual binding sites, such as in lectin polypeptide oligomer. In addition, the geometry of such oligomers helps to distinguish arrangements of cell surface polysaccharides and glycoconjugate crosslinks in some cases [35].

## 5. Extraction and Purification of Lectins

Traditionally, lectins are isolated from their natural sources by directly extracting them using buffer solutions from finely macerated tissues [37]. Depending on the type of sample, it may be frequently necessary to pre-treat the sample using large quantities of acids or organic solvents to remove lipids and other interfering substances [38,39]. Once the crude extracts have been obtained, it is necessary to precipitate the lectins, either using ammonium sulphate or ethanol, before centrifuging the sample to separate the parts of the proteins that are not of interest [4]. The fraction collected may be purified by different techniques, such as gel-filtration chromatography, ion exchange chromatography, hydrophobicity chromatography and mainly affinity chromatography, because of the ability of lectins to selectively bind to sugars [40].

It is important to note that the similarity between isoforms of lectins hinders their complete separation, so they are usually obtained as semi-pure fractions [6]. It must be taken into account that although some of the isoforms only slightly differ in the functional properties, these differences significantly affect their biological activity in other cases. Two isoforms of *Phaseolus vulgaris* lectin (PHA-E and PHA-L) exhibit significant differences regarding their mitogenic effect on leukocytes and in their anti-insecticidal effect. Furthermore, it has been observed that *Phaseolus acutifolius* contains at least two different types of lectins with different activity [41,42,43]. These differences may be due to a large number of factors, such as the imprecise cut-off of the C-terminal mature polypeptides, differential expression in tissues and/or developmental stages and allelic variation when the material is not genetically uniform [6].

The yields of purification of lectins from the previously described techniques are generally low and depend on the source. For example, the highest performance found in animals was 9.4 mg per each 100 g of carp gills (*Hypophthalmichthys nobilis*) [44], while the lowest was 0.35 mg per 100 g of jelly from *Nemopilema nomurai* [45], with an average of 2.92 mg per 100 g of animal tissue. In the case of fungi, the recovery of lectins is very low, so it is considered that the production from these organisms is impractical [40]. These proteins are mainly located in the fruiting bodies, which are multicellular structures of the fungi responsible for producing spores. A yield of 2.6 mg per 100 g has been reported for the fresh fruiting bodies of *Pleurocybella porrigens* [46] and 15.3 mg per 100 g for the dry fruiting bodies of *Inocybe umbrinella* [47]. On the other hand, plants seem to have the best yields of 13–107 mg per 100 g of seed, especially legumes [48,49]. However, in the case of non-leguminous plants, the lectin content is lower, such as the yield of 3.3 mg lectin per 100 g seed from *Hibiscus mutabilis* [50]. Table 2 compiles some examples of lectins purified by traditional techniques and their yields.

## 6. Production of Recombinant Lectins

Obtaining heterologous proteins could facilitate the cost-effective production of bioactive lectins for pharmaceutical purposes [56]. These systems offer higher yields than conventional purification methods, reducing costs and time. Furthermore, it may avoid the problem of contamination with other compounds [15], allowing only a single protein to be obtained. In addition, this methodology solves the problem of separating isoforms by producing a single product with defined properties. This also allows for the study of the relation sequence function by directed mutagenesis [6]. The most important models that have been proposed so far for the heterologous production of lectins are bacteria and yeasts, while other models have also been studied, such as mammalian cells, plants and insects [14]. Each model offers advantages and disadvantages.

### 6.1. Production of Recombinant Lectins in Bacteria

*E. coli* was the first and the most widely used organism to produce recombinant proteins due to many advantages, including its simplicity, the knowledge of its physiology and its genome as well as its rapid rate of duplication [14,57]. However, it is not always the best alternative for the production of certain lectins, due to the lack of intracellular organelles. Thus, the proper processing of these proteins may not occur. Another disadvantage of producing proteins in bacteria is that those that are large in size or expressed in high concentrations tend to form insoluble cytoplasmic aggregates, which are better known as “inclusion bodies” [10,14,58]. In these “inclusion bodies”, the processed protein could be inactivated with structural artefacts added, such as non-native disulphide bonds and free non-habitual cysteines [14]. This problem has been approached by some studies where they propose the processing of inclusion bodies by the solubilisation with denaturing agents, before later renaturing the protein to its active form [8]. It is important to note that despite the limitations, the successful production of eukaryotic lectins in *E. coli* has been reported, where even when the final product is not identical to the native lectin, the biological activity is preserved. *E. coli* was used to produce a lectin of about 14.5 kDa, which is normally expressed in roots of rice (*Oryza sativa* L.) in response to salt stress (SALT) [5]. The resultant lectin maintained its biological activity (agglutination units of 0.078 µg/mL) with a similar molecular weight to the native counterpart. This is an interesting result considering that most of the plant lectins produced in bacteria usually lose their biological activity. In order to obtain more information about the processing of this lectin, we analysed the SALT sequence with the NetNGlyc 1.0 (DTU Bioinformatics, Lyngby, Denmark) and SignalP 4.0 programs (DTU Bioinformatics, Lyngby, Denmark). We found that although it contains a potential site for *N*-glycosylation at position 101, it lacks the ER signal peptide, which suggests that SALT is synthesized in the cytoplasm, where post-translational changes are not produced and therefore, there will be no considerable changes in its biological activity. However, it is important to note that the protein was obtained into inclusion bodies and it was necessary to treat the protein with sodium dodecyl sulphate (SDS) to bring it to its soluble form and later renature it, so the final yield was 14.6 mg of recombinant lectin/L in the culture medium.

As the solubilisation/renaturation technique sometimes results in a significant decrease in yield, new strategies have been proposed to avoid them, such as pH modulation, growing temperatures below the standard and the fusion of the protein with peptides that enhances the expression and solubility. A variety of different peptides have been tested for this purpose and thus far, the SUMO peptide (small ubiquitin-related modifier) has been shown to be the most effective [10,11,12]. In a recent work, the SUMO peptide was coupled to the coding sequence of the ASAL lectin from garlic (*Allium sativum*), which is a mannose-binding glycoprotein with a 25-kDa size. Sixty percent of the protein was excreted in a soluble form, achieving a final production yield of 5 mg of recombinant lectin/L of culture medium. By analysing the ASAL sequence with the NetNGlyc 1.0 and SignalP 4.0 programs, we found that it contains two possible *N*-glycosylation sites in positions 151 and 168 and also contains a signal peptide of 30 amino acids. This suggests that ASAL is synthesized in ER. The authors reported structural and carbohydrate recognition alteration, although these alterations did not affect its biological activity. The modified strains of *E. coli* were obtained in order to avoid such problems where synthetic routes for the addition of eukaryotic *N*-glycosylations [59] and oxidation of disulphide bonds in the cytoplasm were included [60]. The AD494 strain, a K-12 mutant in thioredoxin reductase (trxB) or the Origami strain (also derived from K-12), contains a further mutation in the glutathione reductase (gor) [61].

### 6.2. Production of Recombinant Lectins in Yeast 

Yeasts are suitable organisms for the heterologous expression of eukaryotic proteins, since they combine the easy genetic manipulation and rapid growth of prokaryotic organisms, with a subcellular machinery necessary for post-translational modifications, such as glycosylations, disulphide bonds and proteolytic processing [62]. The molecular understanding of the physiology and genetics of *Saccharomyces cerevisiae* makes it suitable to initially be considered as one of the most promising expression systems for this purpose [62]. However, in the case of glycoproteins, there are multiple drawbacks associated with the use of this model, such as the high antigenicity of their glycosylations [14], accumulation of product within vacuoles and cytoplasm [63,64], incomplete processing of their signalling peptides [63] and poor performance [6]. As a result, *Saccharomyces* shows poor expression of glycoproteins and has been replaced with *Pichia pastoris*, which is capable of producing glycoproteins in high concentrations and also excreting them, allowing the production of larger quantities in the culture medium [64]. *P. pastoris* also offers the possibility of using the methanol-regulated promoter (AOX1) [15] and it does not add α-1-3 mannose residues, which are responsible for the antigenic nature of glycoproteins excreted by *S. cerevisiae* [63,65]. In addition, the *N*-oligosaccharides in *P. pastoris* appears to be similar, at least in size, to the oligosaccharides of higher organisms [66]. In recent years, this discovery has opened up a range of possibilities for the expression of glycoproteins in *P. pastoris* and has proven to be an excellent system to produce some types of lectins that could be directed into a secretory pathway.

*P. pastoris* was used to produce a lectin from *Nicotiana tabacum*, which is better known as “Nictaba” [15]. In its native form, Nictaba is a homodimer of 19 kDa, whose synthesis is carried out only under the induction by jazmonates. The coding sequence was fused to the *S. cereviciae* α-factor, which is a peptide that targets the protein to the ER and allows the protein to be included in the secretory pathway. Although the authors report incomplete α-factor cleavage, the binding activity of the recombinant product was similar to its native counterpart and the final yield was 6 mg of recombinant protein/L of culture medium. We had analysed the sequence of this lectin with the NetNGlyc 1.0 and SignalP 4.0 programs and we found that there is no presence of signal peptide or possible glycosylation sites, so that Nictaba is a lectin that naturally occurs in free polysomes in the cytosol. However, it contains potential glycosylation sites, indicating that *Pichia pastoris* is a good system for obtaining heterologous lectins. However, it is necessary to consider that for lectins produced in the cytosol and include possible glycosylation sequences, the heterologous expression in yeast using signalling peptides, such as α-factor, could represent a disadvantage. This is because the addition of non-habitual *N*-glycosidic antennae could interfere with its activity. In such cases, it is advisable to make point mutations over the sequons to try to avoid aberrant glycosylations. Other studies report incomplete processing of the N-terminal end when α-factor for the excretion of heterologous proteins is added [6,7,14,62,67]. In this sense, there have been other studies that propose new peptides for the successful excretion of proteins into the extracellular space. Two legume lectins (*P. vulgaris* PHA-E and *G. nivalis* GNA) in *P. pastoris* were expressed using the N-terminal phytohemagglutinin of *P. vulgaris* as the signal peptide in order to compare the excretion and post-processing effectiveness in relation to the α-factor of *S. cerevisiae* [6]. The results show that the PHA-E signal peptide directed the protein secretion when they were correctly processed at the N-terminal extension, while proteins secreted under the α-factor peptide control presented heterogeneous N-terminal extensions. This suggests that the PHA-E signal peptide may have a wider utility in the production of recombinant proteins in *Pichia*.

As in bacteria, modified *Pichia* strains are capable of producing eukaryotic glycoproteins. The *Pichia* strain was modified by eliminating four endogenous genes and adding 14 heterologous glycoenzyme genes to allow them to replicate the *N*-glycosylation steps in humans [13]. Recently, some studies have also focused on the engineering of *O*-glycosylation to produce sialylated *O*-linked glycans in yeast [10].

### 6.3. Production of Recombinant Lectins in Plants

The transformation and modern techniques of plant culture allow the production of genetically-modified plants to be used as bioreactors for the production of recombinant proteins and/or industrially relevant metabolites [68]. As eukaryotes, plants provide a suitable environment for protein folding and formation of disulphide bonds, assembly of multimeric proteins and post-translational modifications [69].

The lectin from *Urtica doica* (UDA) was produced in genetically-modified tobacco plants [70]. UDA contains a C-terminal end with vesicle localization labels. For this project, the C-terminal end of the protein was removed to achieve its cellular excretion and its purification. The authors reported that the proposed modifications did not interfere with the correct folding of the lectin, which maintains its antifungal activity. The analysis of the UDA sequence with the SignalP 4.0 program revealed that the lectin contains an ER signal peptide which was not altered in the construct, so the only difference in the synthesis between the native product and the recombinant one was the removal of the tag for vesicular retention [70]. A lectin from *Robinia pseudoacacia* was cloned in transgenic tobacco plants. The obtained lectin maintained the same size and biological activity as its native counterpart and its signal peptide was correctly cleaved. In addition, evidence was presented that suggests the formation of protein oligomers. The final yield was 1 mg of lectin per 50 g of fresh leaves [2].

The disadvantages of lectin production in plants include poor performance, difficulties associated with the generation process compared to the microorganisms, cost and required materials, facilities and the time required. It is also important to consider that when the glycosylation sequons are very close to the CRD, the conformation of the carbohydrates can be directly related to the recognition capacity of their ligands [35]. In these cases, it is suggested to use the same species of origin or some phylogenetically related plant.

### 6.4. Production of Recombinant Lectins in Animal Cells

The manipulation of animal cells is more expensive and complex than plant cells or microorganisms. However, they have the advantage of producing glycoproteins with a similar glycan profile to humans [59], so there is a great interest in the understanding and optimization of these systems. Insect lines have been noted for their relative simplicity, although their glycoproteins typically contain different glycans with respect to mammalian glycans (mainly mannose-rich *N*-glycans and mainly paucimannosidic *N*-glycans). In order to avoid these drawbacks, new strategies have emerged in recent years, such as the introduction of mammalian glycosylases regulated under the action of inducible promoters [71] and CRISPR-Cas technology to modify their glycosylation pathways [72].

Undoubtedly, the system most commonly used for production of recombinant proteins for therapeutic purposes is the Chinese hamster ovary (CHO) cell line, because it represents a very well characterized model and also their glycosylation patterns are very similar to humans. This is particularly important when the lectin of interest has therapeutic purposes and the glycosylations are not only important to maintain its activity, but also interfere with the blood retention time and immune response. Within the reports of human lectins that have been produced in CHO cells and maintain their original properties, the mannan-binding lectin (MBL) stands out. MBL is a C-type animal lectin present in blood serum, which plays an important role in the innate immunity system [73]. This lectin is particularly interesting because it has the ability to neutralize the influenza virus. Both the human and recombinant versions exhibited neutralizing activity against the virus and both prevented viral propagation from infected primary cells to neighbouring cells. The final yield was 128.8 μg/mL of culture medium [73,74,75].

Animal systems can be used for the production of lectins of different origins, although it has been observed that sometimes the products exhibit important structural alterations. Considering the difficulties of their manipulation, these are not widely recommended for the production of lectins from other organisms. The production of a soybean agglutinin (BSA) in BS-C-1 monkey cells was reported to have a final yield of 1 mg protein/L of culture. The specificity of carbohydrate binding was not affected, although the product showed a decrease in its hemagglutinating capacity of at least four times with respect to the native lectin. In addition, it was observed that although the product was glycosylated, the carbohydrates were mainly oligomannosides, while these are normally Man9 (GlcNac) glycosylations in the native lectin [58].

It is known that glycosylation production in animal cells can be affected by bioprocess parameters, such as the cell line [76], cell density, media composition [77], nutrient levels and supplements [78,79], pH and transfection conditions [21,22]. The interactions between these parameters are complex and, therefore, attention should be paid to the development and optimization of this process [23]. In this sense, a recent study reported a new glycoengineering strategy, called GlycoDelete, to access glycoproteins with homogeneous *N*-glycans by shortening the Golgi *N*-glycosylation pathway in mammalian cells [80]. Table 3 shows a summary of the yields obtained for the production of recombinant lectins in different biological systems.

## 7. Summary of Considerations for Recombinant Lectin Production

The strategies for the production of recombinant lectins focus on their biological activity. These strategies must consider the modification of host organisms, aiming to approximate the original synthesis conditions, although sometimes modifications are also needed. An appropriate choice of such strategies is fundamental for the maintenance of the desirable characteristics of the product and for obtaining good yields of production. The most important aspects to consider for these purposes are:

(a) Considerations according to their synthesis

Knowing the site where lectin synthesis is carried out is perhaps the most important issue to be considered for successful heterologous expression. The lectins synthesized in the ER lumen differ to a great extent from those that are synthesized in cytoplasm. If there is no detailed information about its synthesis, we suggest using the SignalP 4.0 program to know if the lectin has a signal peptide to be translocated to ER during translation.

- Lectins synthesized in cytoplasm. The lectins produced by free ribosomes in the cytoplasm, whether or not they have possible glycosylation sites, can be successfully expressed in *E. coli* with the consideration that it is necessary to introduce only the sequence of the mature protein. If the product obtained tends to form inclusion bodies, denaturation/renaturation strategies or the addition of excretion tags might be needed. When the lectin is normally synthesized in the cytoplasm, but contains possible glycosylation sites, and is desired to be expressed in yeast, it may be useful to mutate the sequons to avoid the addition of non-desirable glycosidic antennas. Proteins synthesized in cytoplasm can also be expressed in plants and animal cells, although these alternatives require more complex strategies, more investment of time and money. Furthermore, these generate lower yields than bacteria and yeasts. When using these strategies, it may be advisable not to add signal peptides that redirect the synthesis of the lectin in question to the ER and if added, it is recommended to mutate possible glycosylation sites to avoid the addition of non-desirable glycosidic antennas.
- Lectins synthesized in ER. Those lectins that are commonly synthesized in ER but do not contain possible glycosylation sites can also be expressed in *E. coli* with the previously mentioned recommendations. However, it is important to consider that if an oxidizing or chaperoning environment is required for the correct folding, it may be convenient to use a modified strain of *E. coli* with non-reducing cytoplasm and chaperones. Yeasts may be a better alternative. It is important to note that in these cases, no drawbacks caused by non-native glycosylations will occur because the lectin will not contain any sequons. If the protein is normally synthesized in ER and if it contains sequons, we do not recommend using *E. coli* since the product will not contain glycosylations. Although it was shown that it is possible to obtain this type of lectins when using bacteria as producing organisms in some of the reports, all showed some type of structural alteration and/or partial loss of their functions. Therefore, it is preferable to use yeasts or higher organisms since they contain all the post-translational machinery, chaperones and the ER with an oxidizing environment. These lectins can also be expressed in plants and animal cells. In such cases, signalling peptides must be added, which can be recognized by the host organism and it is not necessary to mutate possible glycosylation sites.

(b) Considerations according to their origin

**Figure 1:**
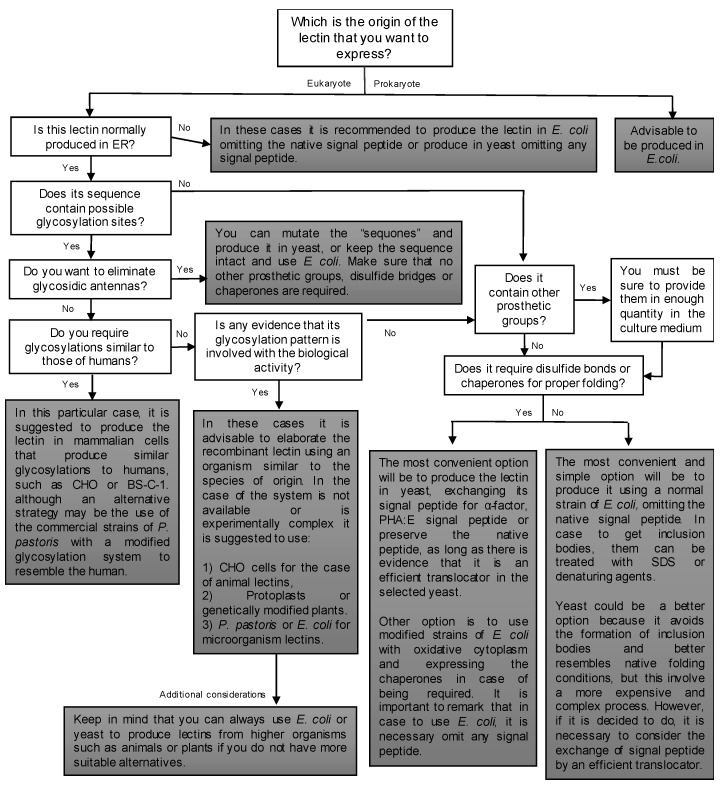
Diagram of strategies for the successful production of recombinant lectins according to their characteristics and origin.

The lectins of archea and bacteria do not represent a problem in the choice of the generating organism as there is the option of changing to other systems, such as bacteria and yeasts. In the case of vegetable lectins, the production in microorganisms is always favourable due to the high yields and simplicity of manipulation. In case of not being a feasible alternative, it is possible to use well-established tobacco-like models, except when the glycosylation sequences are very close to the CRD. The conformation of the carbohydrates can influence the recognition capacity of their ligands, so in these cases, we suggest that the same species of origin or some phylogenetically-related plant should be used. Lectins of animal origin may also be produced in plants, although the antigenicity of carbohydrate antennas should be considered for pharmaceutical purposes. Figure 1 shows a scheme of the main considerations.

## 8. Final Remarks

Glycobiology comprises an extensive and scarcely explored world that promises to be very useful for future scientific development. In this sense, lectins play a fundamental role in the discovery of new drugs and materials that can contribute to studying and better understanding some biological processes that are influenced by the action of carbohydrates. However, the complexity of these proteins has greatly hindered their large-scale production. It is necessary to understand that in the particular case of lectins, the protein does not adapt to the system and thus, the system must be adapted to the protein. Understanding the needs of each lectin involves essentially trying to find the strategy that provides higher yields and guarantees the functionality of the product. This review aims to show the complex world of lectins by analysing their functions, characteristics and synthesis process, as well as showing some basic bioinformatic tools that can be used to obtain greater information. The information provides examples of some of the different strategies that have been developed to produce recombinant lectins in order to maintain their biological activity. These strategies focus on the modification of host organisms in order to approximate the original synthesis conditions, although it is also sometimes necessary to modify the polypeptide sequences. Being a heterogeneous group of proteins, it is not possible to declare the superiority of any of the systems explored for the heterologous production of lectins. However, the information is integrated into a complete analysis in this present paper, which can serve as a guide for choosing the most appropriate strategy according to the particular characteristics of each lectin. The recommendations here presented have been proposed taking simplicity as a priority, but also provide the option of more complex alternatives.

## Figures and Tables

**Table 1:** The overall view of lectins from different organisms.

Parameter	Organism	Lectin Family	Reference
Cell localization	All	Extracellular lectins, intracellular endoplasmic reticulum (ER) lectins, Golgi lectins, cytoplasmic lectins, membrane-bound lectins.	[30]
Structural and evolutionary sequence similarities	All	Beta prism lectins (B-type), calcium dependent lectins (C-type), lectins with Ficolins-Fibrinogen/collagen domain (F-type), garlic and snow drop lectins (G-type), hyaluronan bonding proteins or hyal-adherins (H-type), immunoglobulin superfamily lectins (I-type), jocob and related lectins (J-type), legume seed lectins (L-type), alpha mannosidase related lectins (M-type), nucleotide phosphohydrolases lectins (N-type), ricin lectins (R-type), *Tachypleus tridentatus* (T-type), wheat germ agglutinin (W type), Xenopus egg lectins (X type)	[33]
Taxonomic origin	All	Plants lectins, animal lectins, microbial lectins.	[30]
Carbohydrate-specificities	Plant and animals	d-mannose (d-glucose)-binding lectins, 2-acetamido-2-deoxy-glucose-binding lectins, 2-acetamido-2-deoxy-galactose-binding lectins, d-galactose-binding lectins, l-fucose-binding lectins, other lectins.	[34]
	All	Glucose/mannose-binding lectins, galactose and *N*-acetyl-d-galactosamine-binding lectins, l-fucose-binding lectins, sialic acids-binding lectins.	[33]
Function	Microbial	Hemagglutinins, adhesins, and toxins.	[30]
	Animal	Galectins, selectins, collectins and pentraxins.	[32]
Structure	Animals	C-type, galectins, P-type (M-6-PR), I-type, pentraxins, heparyn binding type, F-type, calnexin, M-type, L-type, R-type, F-box, ficolin, chitinase-like.	[32]
	Plants	Amaranthins, nictaba related proteins, heveins (chitin binding lectins), jackalins, legume lectins, *Galanthus nivalis* agglutinin and GNA-related lectins, monocot mannose binding lectins, and plant lectins with Ricin-B domain.	[31]

**Table 2:** Yields of different lectins obtained by traditional purification techniques.

Natural Source	Purification	Lectin Yield	References
*Acropora millepora* (coral), plasma fluid.	Mannose affinity chromatography.	0.7 mg/100 mL of plasma	[51]
*Aristichthys nobilis* (bighead carp), gills.	Chromatography on diethylaminoethanol (DEAE)-Sepharose, Sephacryl S-200 and superdex-200.	9.4 mg/100 g	[44]
*Pleurocybella porrigens.*	Chromatography on Sepharose 4B, BioAssist Q.	2.6 mg/100 g	[46]
*Bubalus bubalis* (buffalo), heart tissue.	Ammonium sulphate precipitation and chromatography on Sephadex G50.	0.97 mg/100 g	[52]
*Hibiscus mutabilis.*	Ion exchange chromatography on SP-Sepharose and gel filtration in Superdex 75 and Superdex 200.	4.04 mg/100 g	[50]
*Inocybe umbrinella* (*mushroom*).	Ion exchange chromatography on DEAE cellulose and carboxymethylcellulose, and gel filtration on Superdex 75.	15.3 mg/100 g	[47]
*Holothuria scabra* (sea cucumber), coelomic fluid.	Ultrafiltration and chromatography on Phenyl-Sepharose.	1.6 mg/100 g	[53]
*Macoma birmanica* (marine bivalve), foot muscles.	Ammonium sulphate precipitation and chromatography on *N*-acetylglucosamine Sepharose 4B.	4.5 mg/100 g	[54]
*Nemopilema nomurai* (jellyfish).	Chromatography on SP-Sepharose and BSM-Toyopearl.	0.35 mg/100 g	[45]
*Phaseolus vulgaris* (common bean), cultivar french bean.	Chromatography on SP-Sepharose, Affi-gel blue, Q-Sepharose, and Superdex 200.	4.8 mg/100 g	[55]
*Phaseolus vulgaris* (common bean), cultivar dark red kidney bean	Chromatography on DEAE-cellulose and Affi-gel blue gel.	107 mg/100 g	[49]
*Phaseolus vulgaris* (common bean) cultivar Anasazi bean	Affi-gel blue gel, Mono S and chromatography on Superdex 200.	13 mg/100 g	[48]

**Table 3:** Yields of different lectins obtained by recombinant techniques.

Lectin	Natural Source	Producing Organism	Production Yield	References
SALT	*Oryza sativa L*	*E. coli*	14.6 mg/L	[5]
ASAL	*Allium sativum*	*E. coli*	5 mg/L	[11]
*Artocarpus incisa* lectin	*Artocampus incisa*	*E. coli*	16 mg/L	[81]
NICTABA	*Nicotiana tabacum*	*P. pastoris*	6 mg/L	[15]
PHA-E	*Phaseolus vulgaris*	*P. pastoris*	0.4–1 mg/L	[6]
GNA	*Galanthus nivalis*	*P. pastoris*	1–2 mg/L	[6]
SBA	*Glycine max*	BS-C-1 cells	1 mg/L	[58]
PHA	*Phaseolus vulgaris*	*P. pastoris*	100 mg/L	[82]
GNA	*Galantus nivalis*	*P. pastoris*	80 mg/L	[82]
UDA	*Urtica doica*	*Nicotiana tabacum*	No reported	[70]
*R. pseudoacacia* lectin	*Robinia pseudoacacia*	*Nicotiana tabacum*	2 mg/100 g of dry leaves	[2]
MLB	*Homo sapiens*	Chinese hamster ovary cells	128 µg/mL	[73]
MoL	*Moringa oleifera*	*P. pastoris*	520 mg/L	[83]
MBL	*Gallus Gallus*	HeLa R19 Cells	1.5–2 mg/L	[84]
